# Crystal structures of (*E*)-2-amino-4-methyl­sulfanyl-6-oxo-1-(1-phenyl­ethyl­idene­amino)-1,6-di­hydro­pyrimidine-5-carbo­nitrile and (*E*)-2-amino-4-methyl­sulfanyl-6-oxo-1-[1-(pyridin-2-yl)ethyl­idene­amino]-1,6-di­hydro­pyrimidine-5-carbo­nitrile

**DOI:** 10.1107/S2056989021004126

**Published:** 2021-04-23

**Authors:** Reham A. Mohamed-Ezzat, Galal H. Elgemeie, Peter G. Jones

**Affiliations:** a Chemistry of Natural & Microbial Products Department, National Research Center, Cairo, Egypt; b Chemistry Department, Faculty of Science, Helwan University, Cairo, Egypt; c Institut für Anorganische und Analytische Chemie, Technische Universität Braunschweig, Hagenring 30, D-38106 Braunschweig, Germany

**Keywords:** crystal structure, pyrimidino­nes, hydrogen bonds

## Abstract

The title compounds **3a**, C_14_H_13_N_5_OS, and **3b**, C_13_H_12_N_6_OS, both show an *E* configuration about the N=C bond and a planar NH_2_ group. The mol­ecules, which only differ in the presence of a phenyl (in **3a**) or pyridyl (in **3b**) substituent, are closely similar except for the different orientations of these groups. The amino hydrogen atoms form classical hydrogen bonds; in **3a** the acceptors are the oxygen atom and the cyano nitro­gen atom, leading to ribbons of mol­ecules parallel to the *b* axis, whereas in **3b** the acceptors are the oxygen atom and the pyridyl nitro­gen, leading to a layer structure perpendicular to (01).

## Abstract 2

The title compounds differ in the orientations of the phenyl (in **3a**) or pyridyl (in **3b**) groups. Classical hydrogen bonds involving the amino group lead to one- or two-dimensional packing patterns, respectively.

## Chemical context   

Dimethyl *N*-cyano­dithio­imino­carbonate (**2**) is an important starting material for the synthesis of various classes of heterocycles (Elgemeie & Mohamed, 2014 ▸), *e.g.* azoles, azines and azoloazines (Thomae *et al.*, 2009 ▸). It has been used effectively in the synthesis of a range of anti­bacterial (Paget *et al.*, 2006 ▸), anti­cancer (Hu *et al.*, 2014 ▸) and other biologically significant products (Marsault *et al.*, 2007 ▸).

Pyrimidino­nes are multipurpose heterocyclic compounds that are common in nucleic acids and find diverse applications in drug planning (Elgemeie *et al.*, 2019 ▸; Elgemeie & Mohamed, 2019 ▸); they are important in pharmaceutical chemistry because of their pharmacological potential (Galmarini *et al.*, 2003 ▸). Research in the pharmaceutical chemistry of pyrimidone derivatives has become an active field, since several pyrimidinone-based compounds have been extensively used as clinical drugs to treat numerous types of viruses with high therapeutic effectiveness (Simons *et al.*, 2005 ▸); their biotic profile and synthetic availability have been attractive in their design and development as possible chemotherapeutics. In particular, pyrimid­inone derivatives have recently become significant in the improvement of anti-coronavirus agents (Pruijssers *et al.*, 2019 ▸).

In order to access this class of compounds, a variety of new synthetic methods has been developed (Xu *et al.*, 2004 ▸). Recently, we have designed the syntheses of several pyrim­idinone derivatives starting from activated nitriles (Elgemeie *et al.*, 2015*a* ▸,*b* ▸; Abu-Zaied *et al.*, 2020 ▸, 2021 ▸). As part of this program, the reactions of 2-cyano-*N*′-(1-phenyl­ethyl­id­ene)acetohydrazide (**1a**) or 2-cyano-*N*′-(1-(pyridin-2-yl)ethyl­idene)acetohydrazide (**1b**) with **2** in KOH/EtOH were studied. These reactions gave products that were crystallized from DMF and identified by X-ray crystallography as the title compounds (**3a**,**b**). ^1^H NMR spectra of **3a** showed SCH_3_ protons at δ 2.55 ppm and the free NH_2_ protons at δ 8.52 ppm. The formation of **3** from **1** and **2** is assumed to proceed *via* initial addition of the active methyl­ene group of **1** to the double bond of **2**, followed by elimination of CH_3_SH and cyclization *via* addition of the NH group to the cyano group.
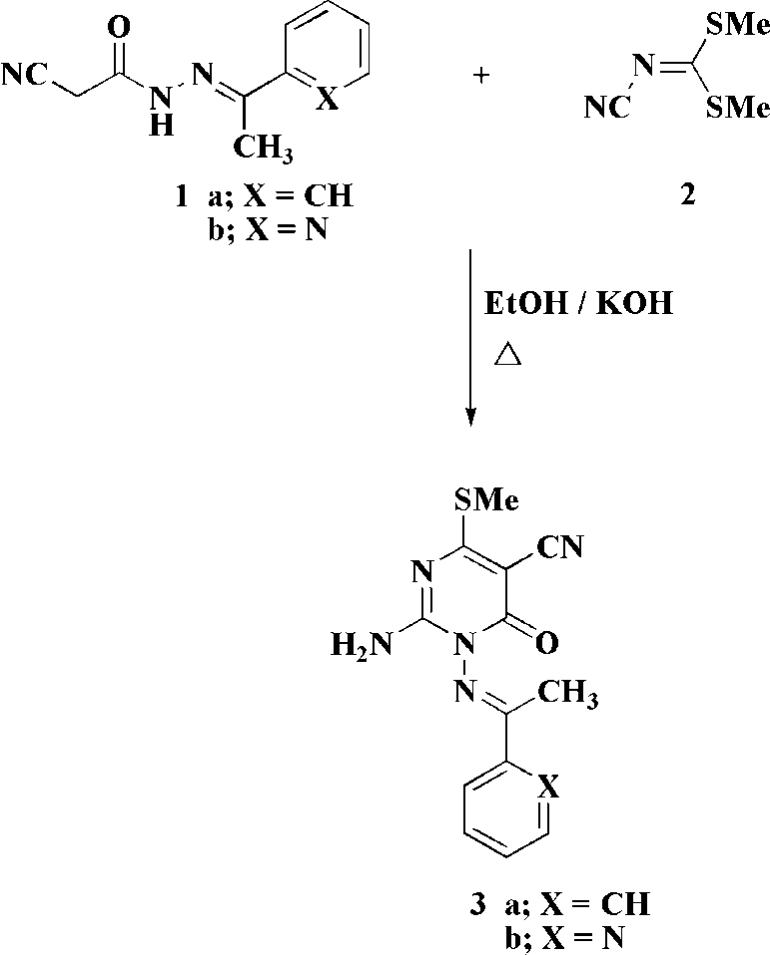


## Structural commentary   

**Figure 1:**
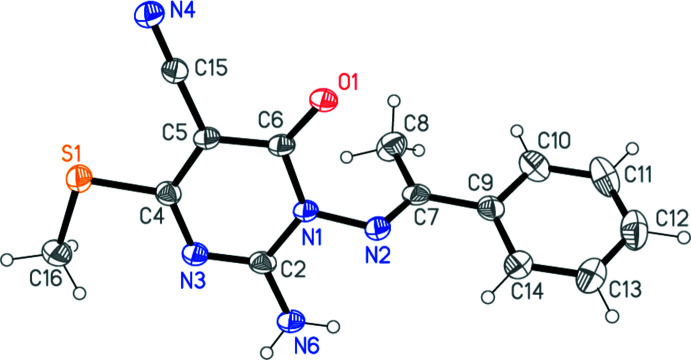
The structure of compound **3a** in the crystal. Ellipsoids correspond to 50% probability levels.

The structure determinations confirm the expected chemical structures of **3a** and **3b**; the respective mol­ecules are shown in Figs. 1 ▸ and 2 ▸. In both compounds, the configuration about the double bond N2=C7 is *E* and the amino group is planar. The pyrimidine ring dimensions are closely similar; *e.g*. the shortest bonds are C2—N3, the narrowest angles are at C6 (which bears the oxo substituent) and the widest angles are at C4 (which bears the methyl­thio group). These and a selection of other dimensions are presented in Tables 1 ▸ and 2 ▸.

**Figure 3:**
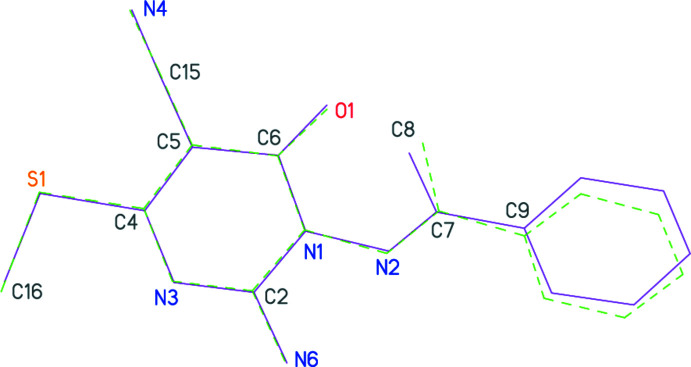
A least-squares fit of the mol­ecules of **3a** (solid bonds) and **3b** (dashed bonds; mol­ecule inverted with respect to the deposited coordinates). Only the fitted atoms are labelled; their r.m.s. deviation is 0.16 Å.

The compounds differ chemically only in the phen­yl/pyridyl substituents. A least-squares fit of the two mol­ecules shows a moderate difference in the orientation of these groups (Fig. 3 ▸, Tables 1 ▸ and 2 ▸); this may be associated with the role of the pyridyl nitro­gen as a hydrogen-bond acceptor in **3b** (see below).

Whereas the immediate substituent atoms of the pyrimidine rings lie close to the ring plane for **3a** [maximum deviation of 0.103 (2) Å for N6], the substituents O1 and C15 of **3b** are more significantly displaced [by 0.203 (2) and 0.179 (3) Å, respectively, to the same side of the ring]. The inter­planar angles between the six-membered rings are 56.49 (6)° for **3a** and 63.12 (3)° for **3b**.

Intra­molecular hydrogen bonds N6—H062⋯N2 (not shown explicitly in Figs. 1 ▸ and 2 ▸) are observed in both mol­ecules (Tables 3 ▸ and 4 ▸).

## Supra­molecular features   

**Figure 4:**
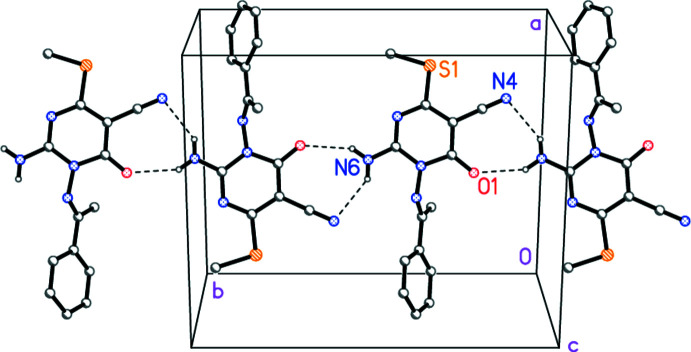
Packing diagram of compound **3a** viewed perpendicular to (102) in the region *z* ≃ 0.25. Dashed lines indicate classical hydrogen bonds. Hydrogen atoms not involved in such bonds are omitted for clarity.

**Figure 5:**
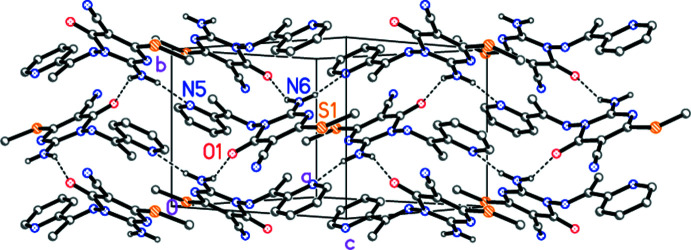
Packing diagram of compound **3b** viewed perpendicular to (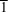
01). Dashed lines indicate classical hydrogen bonds. Hydrogen atoms not involved in such bonds are omitted for clarity.

In both structures, the hydrogen atoms of the amino groups act as hydrogen bond donors (Tables 3 ▸ and 4 ▸). In **3a**, neighbouring mol­ecules are connected *via* the same 2_1_ operator, leading to ribbons of mol­ecules parallel to the *b* axis (Fig. 4 ▸). In **3b**, one hydrogen bond is formed *via* a 2_1_ and one *via* an *n* glide operator, leading to layers parallel to (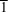
01) (Fig. 5 ▸).

## Database survey   

A search of the Cambridge Database (ConQuest Version 2.0.5) for 6-oxo­pyrimidines with the same substitution pattern (N at C2, S at C4, cyano at C5 and N at N1) revealed only our previous structures (Elgemeie *et al.*, 2015*a* ▸,*b* ▸; refcodes WUSMAA and WUSMUU); the substituents at N1 were NH-SO_2_-*p*-C_6_H_4_Br and N=CH-2-tht, respectively.

## Synthesis and crystallization   

**General procedure for the synthesis of compounds 3:** A mixture of the appropriate 2-cyano-*N*′-(1-aryl­ethyl­idene)acetohydrazide (**1**) (0.01 mol), dimethyl *N*-cyano­dithio­imino­carbonate (**2**) (0.01 mol) and anhydrous potassium hydroxide (0.01 mol) was refluxed in ethanol (10 mL). The reaction mixture was then poured onto ice–water; the solid product thus formed was filtered off and recrystallized from DMF.

**3a**: According to the general procedure, 2-cyano-*N*′-(1-phenyl­ethyl­idene)acetohydrazide (**1a**) was refluxed with **2** for 3 h. Compound **3a** was afforded as a pale-yellow solid (92%); m.p. 498–501 K; IR (cm^−1^) υ 3719 and 3437 (NH_2_), 2202 (CN) and 1657 (C=O). ^1^H NMR (400 MHz, DMSO-*d*_6_): δ 2.20 (*s*, 3H, CH_3_), 2.55 (*s*, 3H, SCH_3_), 8.52 (*s*, *br*, 2H, NH_2_), 8.045–8.065 (*d*, *J* = 8 Hz, 2H, 2 CH), 7.59–7.62 (*m*, 1H, CH), 7.50–7.54 (*m*, 2H, 2 CH). Analysis calculated for C_14_H_13_N_5_OS (299.35): C, 56.17; H, 4.38; N, 23.40; O, 5.34; S, 10.71%. Found: C, 55.89; H, 4.25; N, 23.15; S, 10.52%.

**3b**: According to the general procedure, 2-cyano-*N*′-(1-(pyridin-2-yl)ethyl­idene)acetohydrazide (**1b**) was refluxed with **2** for 30 min. Compound **3b** was afforded as a buff solid (80%); m.p. 649–652 K; IR (cm^−1^) υ 3774 (NH_2_), 2172 (CN) and 1635 (C=O). ^1^H NMR (400 MHz, DMSO-*d*_6_): δ 2.20 (*s*, 3H, CH_3_), 2.51 (*s*, 3H, SCH_3_), 8.54 (*s*, *br*, 2H, NH_2_), 8.73–8.74 (*d*, *J* = 4 Hz, 1H, CH), 8.29–8.31 (*d*, *J* = 8 Hz, 1H, CH), 7.95–8.00 (*t*, 1H, CH); 7.59–7.63 (*t*, *J* = 8 Hz, 1H, CH). Analysis calculated for C_13_H_12_N_6_OS (300.34): C, 51.99; H, 4.03; N, 27.98; O, 5.33; S, 10.68. Found: C, 51.73; H, 4.22; N, 27.71; S, 10.39%.

Crystals of **3a** proved to be almost all twinned, by 180° rotation about c*. Data were collected from a twinned crystal, but the refinement using the ‘HKLF 5’ method was no better than satisfactory (*wR*_2_ *ca* 0.11). Finally, an untwinned crystal was discovered. Despite its less regular reflection shape, the results proved to be slightly better in terms of the *wR*_2_ value, and the results quoted here are for this untwinned crystal.

## Refinement   

Crystal data, data collection and structure refinement details are summarized in Table 5 ▸. The NH hydrogen atoms were refined freely. The methyl groups were refined as idealized rigid groups allowed to rotate but not tip (AFIX 137; C—H 0.98 Å, H—C—H 109.5 °). The hydrogens of the aromatic rings were included using a riding model starting from calculated positions (C—H_aromatic_ 0.95 Å). The *U*(H) values were fixed at 1.5 (for the methyl H) or 1.2 times the equivalent *U*_iso_ value of the parent carbon atoms.

## Supplementary Material

Crystal structure: contains datablock(s) 3a, 3b, global. DOI: 10.1107/S2056989021004126/jy2007sup1.cif

Structure factors: contains datablock(s) 3a. DOI: 10.1107/S2056989021004126/jy20073asup2.hkl

Structure factors: contains datablock(s) 3b. DOI: 10.1107/S2056989021004126/jy20073bsup3.hkl

Supporting information file. DOI: 10.1107/S2056989021004126/jy20073asup4.cml

CCDC references: 2078221, 2078220

Additional supporting information:  crystallographic information; 3D view; checkCIF report

## Figures and Tables

**Figure 2:**
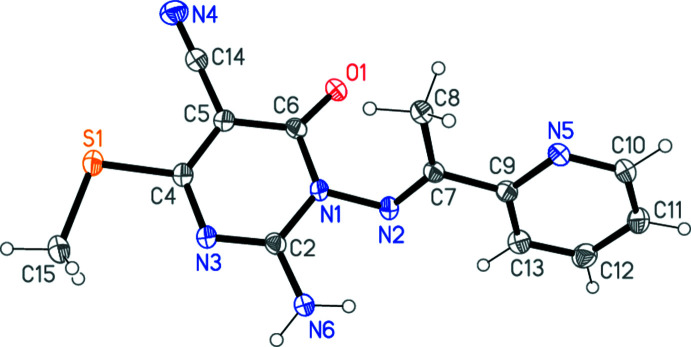
The structure of compound **3b** in the crystal. Ellipsoids correspond to 50% probability levels.

**Table 1:** Selected geometric parameters (Å, °) for **3a** [Chem scheme1]

N1—N2	1.4248 (17)	C4—S1	1.7522 (16)
C2—N3	1.333 (2)	S1—C16	1.7918 (19)
			
C7—N2—N1	114.40 (13)	C4—S1—C16	101.93 (8)
N3—C4—C5	124.02 (14)	N1—C6—C5	112.55 (12)
			
N1—N2—C7—C9	176.69 (12)	N2—C7—C9—C14	12.1 (2)
N2—C7—C9—C10	−166.23 (15)		

**Table 2:** Selected geometric parameters (Å, °) for **3b** [Chem scheme1]

N1—N2	1.4191 (16)	C4—S1	1.7473 (14)
C2—N3	1.3286 (17)	S1—C15	1.8023 (16)
			
C7—N2—N1	115.53 (11)	C4—S1—C15	102.47 (7)
N3—C4—C5	124.29 (12)	N1—C6—C5	112.65 (11)
			
N1—N2—C7—C9	−177.94 (10)	N2—C7—C9—C13	1.27 (18)
N2—C7—C9—N5	−178.88 (12)		

**Table 3:** Hydrogen-bond geometry (Å, °) for **3a** [Chem scheme1]

*D*—H⋯*A*	*D*—H	H⋯*A*	*D*⋯*A*	*D*—H⋯*A*
N6—H061⋯O1^i^	0.84 (2)	2.24 (3)	2.9899 (17)	149 (2)
N6—H062⋯N4^i^	0.84 (2)	2.33 (2)	3.054 (2)	144 (2)
C8—H8*A*⋯O1^ii^	0.98	2.52	3.279 (2)	134
N6—H062⋯N2	0.84 (2)	2.25 (2)	2.6273 (19)	108 (2)

Symmetry codes: (i) -x+1, y+{\script{1\over 2}}, -z+{\script{1\over 2}}; (ii) x, -y+{\script{1\over 2}}, z-{\script{1\over 2}}.

**Table 4:** Hydrogen-bond geometry (Å, °) for **3b** [Chem scheme1]

*D*—H⋯*A*	*D*—H	H⋯*A*	*D*⋯*A*	*D*—H⋯*A*
N6—H061⋯N5^i^	0.86 (2)	2.26 (2)	3.0122 (17)	146.2 (17)
N6—H062⋯O1^ii^	0.853 (19)	2.307 (19)	3.0886 (15)	152.4 (17)
C10—H10⋯O1^iii^	0.95	2.40	3.2351 (17)	147
N6—H062⋯N2	0.853 (19)	2.228 (18)	2.6091 (17)	107.1 (14)

Symmetry codes: (i) x+{\script{1\over 2}}, -y+{\script{3\over 2}}, z+{\script{1\over 2}}; (ii) -x+{\script{1\over 2}}, y+{\script{1\over 2}}, -z+{\script{1\over 2}}; (iii) -x, -y+1, -z.

**Table 5:** Experimental details

	**3a**	**3b**
Crystal data		
Chemical formula	C_14_H_13_N_5_OS	C_13_H_12_N_6_OS
*M*_r_	299.35	300.35
Crystal system, space group	Monoclinic, *P*2_1_/*c*	Monoclinic, *P*2_1_/*n*
Temperature (K)	100	100
*a*, *b*, *c* (Å)	12.15369 (18), 14.9466 (2), 7.68691 (16)	13.4774 (5), 7.6797 (3), 14.2755 (6)
β (°)	91.7607 (16)	112.401 (5)
*V* (Å^3^)	1395.72 (4)	1366.04 (10)
*Z*	4	4
Radiation type	Cu *K*α	Cu *K*α
μ (mm^−1^)	2.12	2.19
Crystal size (mm)	0.2 × 0.2 × 0.02	0.12 × 0.08 × 0.02

Data collection		
Diffractometer	Rigaku XtaLAB Synergy, Single source at home/near, HyPix	Rigaku XtaLAB Synergy, Single source at home/near, HyPix
Absorption correction	Multi-scan (*CrysAlis PRO*; Rigaku OD, 2021 ▸)	Multi-scan (*CrysAlis PRO*; Rigaku OD, 2021 ▸)
*T*_min_, *T*_max_	0.636, 1.000	0.782, 1.000
No. of measured, independent and observed [*I* > 2σ(*I*)] reflections	104992, 2958, 2842	54131, 2905, 2813
*R*_int_	0.061	0.042
(sin θ/λ)_max_ (Å^−1^)	0.634	0.633

Refinement		
*R*[*F* ^2^ > 2σ(*F* ^2^)], *wR*(*F* ^2^), *S*	0.040, 0.106, 1.06	0.036, 0.097, 1.07
No. of reflections	2958	2905
No. of parameters	200	200
H-atom treatment	H atoms treated by a mixture of independent and constrained refinement	H atoms treated by a mixture of independent and constrained refinement
Δρ_max_, Δρ_min_ (e Å^−3^)	0.40, −0.35	0.33, −0.39

Computer programs: *CrysAlis PRO* (Rigaku OD, 2021 ▸), *SHELXT* (Sheldrick, 2015*a* ▸), *SHELXL2018/3* (Sheldrick, 2015*b* ▸) and *XP* (Siemens, 1994 ▸).

## supplementary crystallographic information

### (E)-2-Amino-4-methylsulfanyl-6-oxo-1-(1-phenylethylideneamino)-1,6-dihydropyrimidine-5-carbonitrile (3a) Crystal data

**Table:**

C_14_H_13_N_5_OS	*F*(000) = 624
*M**_r_* = 299.35	*D*_x_ = 1.425 Mg m^−^^3^
Monoclinic, *P*2_1_/*c*	Cu *K*α radiation, λ = 1.54184 Å
*a* = 12.15369 (18) Å	Cell parameters from 58984 reflections
*b* = 14.9466 (2) Å	θ = 3.6–77.1°
*c* = 7.68691 (16) Å	µ = 2.12 mm^−^^1^
β = 91.7607 (16)°	*T* = 100 K
*V* = 1395.72 (4) Å^3^	Plate, pale yellow
*Z* = 4	0.2 × 0.2 × 0.02 mm

### (E)-2-Amino-4-methylsulfanyl-6-oxo-1-(1-phenylethylideneamino)-1,6-dihydropyrimidine-5-carbonitrile (3a) Data collection

**Table:**

Rigaku XtaLAB Synergy, Single source at home/near, HyPix diffractometer	2958 independent reflections
Radiation source: micro-focus sealed X-ray tube	2842 reflections with *I* > 2σ(*I*)
Detector resolution: 10.0000 pixels mm^-1^	*R*_int_ = 0.061
ω scans	θ_max_ = 77.7°, θ_min_ = 3.6°
Absorption correction: multi-scan (CrysAlisPro; Rigaku OD, 2021)	*h* = −15→15
*T*_min_ = 0.636, *T*_max_ = 1.000	*k* = −18→18
104992 measured reflections	*l* = −9→9

### (E)-2-Amino-4-methylsulfanyl-6-oxo-1-(1-phenylethylideneamino)-1,6-dihydropyrimidine-5-carbonitrile (3a) Refinement

**Table:**

Refinement on *F*^2^	Primary atom site location: dual
Least-squares matrix: full	Hydrogen site location: mixed
*R*[*F*^2^ > 2σ(*F*^2^)] = 0.040	H atoms treated by a mixture of independent and constrained refinement
*wR*(*F*^2^) = 0.106	*w* = 1/[σ^2^(*F*_o_^2^) + (0.0516*P*)^2^ + 0.8822*P*] where *P* = (*F*_o_^2^ + 2*F*_c_^2^)/3
*S* = 1.06	(Δ/σ)_max_ = 0.001
2958 reflections	Δρ_max_ = 0.40 e Å^−^^3^
200 parameters	Δρ_min_ = −0.35 e Å^−^^3^
0 restraints	

### (E)-2-Amino-4-methylsulfanyl-6-oxo-1-(1-phenylethylideneamino)-1,6-dihydropyrimidine-5-carbonitrile (3a) Special details

**Table:**

Geometry. All esds (except the esd in the dihedral angle between two l.s. planes) are estimated using the full covariance matrix. The cell esds are taken into account individually in the estimation of esds in distances, angles and torsion angles; correlations between esds in cell parameters are only used when they are defined by crystal symmetry. An approximate (isotropic) treatment of cell esds is used for estimating esds involving l.s. planes. Least-squares planes (x,y,z in crystal coordinates) and deviations from them (* indicates atom used to define plane) 5.1729 (0.0067) x - 0.7702 (0.0093) y + 6.8408 (0.0022) z = 3.9095 (0.0048) * -0.0400 (0.0011) N1 * 0.0328 (0.0011) C2 * -0.0019 (0.0010) N3 * -0.0193 (0.0011) C4 * 0.0105 (0.0011) C5 * 0.0178 (0.0010) C6 0.0930 (0.0023) N2 0.1026 (0.0023) N6 -0.0462 (0.0020) S1 -0.0028 (0.0025) C15 0.0557 (0.0021) O1 Rms deviation of fitted atoms = 0.0241 - 2.6471 (0.0093) x + 12.8703 (0.0066) y - 3.4786 (0.0058) z = 3.0019 (0.0051) Angle to previous plane (with approximate esd) = 56.485 ( 0.063 ) * -0.0047 (0.0012) C9 * 0.0009 (0.0013) C10 * 0.0043 (0.0015) C11 * -0.0056 (0.0015) C12 * 0.0016 (0.0015) C13 * 0.0034 (0.0013) C14 Rms deviation of fitted atoms = 0.0038

### (E)-2-Amino-4-methylsulfanyl-6-oxo-1-(1-phenylethylideneamino)-1,6-dihydropyrimidine-5-carbonitrile (3a) Fractional atomic coordinates and isotropic or equivalent isotropic displacement parameters (Å^2^)

**Table:**

	*x*	*y*	*z*	*U*_iso_*/*U*_eq_	
N1	0.47431 (10)	0.36271 (8)	0.24782 (18)	0.0248 (3)	
N2	0.37697 (11)	0.37729 (8)	0.34251 (18)	0.0264 (3)	
C2	0.53906 (13)	0.43583 (10)	0.2177 (2)	0.0253 (3)	
N6	0.50093 (13)	0.51489 (9)	0.2657 (2)	0.0299 (3)	
H061	0.5387 (18)	0.5609 (17)	0.251 (3)	0.043 (6)*	
H062	0.441 (2)	0.5174 (16)	0.319 (3)	0.050 (7)*	
N3	0.63513 (11)	0.43021 (8)	0.13939 (17)	0.0252 (3)	
C4	0.67045 (12)	0.34825 (10)	0.1009 (2)	0.0240 (3)	
S1	0.79679 (3)	0.33529 (3)	−0.00002 (6)	0.03105 (14)	
C5	0.61368 (12)	0.26976 (9)	0.1394 (2)	0.0238 (3)	
C6	0.50952 (13)	0.27387 (9)	0.2196 (2)	0.0241 (3)	
O1	0.45193 (9)	0.21063 (7)	0.26161 (15)	0.0286 (3)	
C7	0.28666 (13)	0.35750 (9)	0.2578 (2)	0.0260 (3)	
C8	0.27945 (15)	0.32587 (13)	0.0737 (2)	0.0376 (4)	
H8A	0.343475	0.347475	0.011570	0.056*	
H8B	0.211966	0.349048	0.017032	0.056*	
H8C	0.278191	0.260323	0.071499	0.056*	
C9	0.18442 (13)	0.36712 (10)	0.3563 (2)	0.0275 (3)	
C10	0.08665 (16)	0.33019 (13)	0.2925 (3)	0.0387 (4)	
H10	0.084914	0.300421	0.183268	0.046*	
C11	−0.00923 (16)	0.33629 (15)	0.3871 (3)	0.0472 (5)	
H11	−0.075770	0.310997	0.341450	0.057*	
C12	−0.00802 (16)	0.37845 (15)	0.5449 (3)	0.0459 (5)	
H12	−0.073084	0.381751	0.610018	0.055*	
C13	0.08929 (16)	0.41639 (15)	0.6092 (3)	0.0458 (5)	
H13	0.090130	0.446323	0.718242	0.055*	
C14	0.18491 (15)	0.41128 (12)	0.5170 (2)	0.0351 (4)	
H14	0.250819	0.437667	0.562552	0.042*	
N4	0.68678 (12)	0.11352 (9)	0.0616 (2)	0.0315 (3)	
C15	0.65497 (12)	0.18367 (10)	0.0965 (2)	0.0256 (3)	
C16	0.83220 (16)	0.44877 (12)	−0.0485 (3)	0.0419 (4)	
H16A	0.792700	0.467742	−0.155236	0.063*	
H16B	0.911681	0.453120	−0.064728	0.063*	
H16C	0.811591	0.487512	0.048102	0.063*	

### (E)-2-Amino-4-methylsulfanyl-6-oxo-1-(1-phenylethylideneamino)-1,6-dihydropyrimidine-5-carbonitrile (3a) Atomic displacement parameters (Å^2^)

**Table:**

	*U*^11^	*U*^22^	*U*^33^	*U*^12^	*U*^13^	*U*^23^
N1	0.0263 (6)	0.0142 (6)	0.0343 (7)	0.0000 (5)	0.0071 (5)	−0.0012 (5)
N2	0.0272 (6)	0.0182 (6)	0.0342 (7)	0.0009 (5)	0.0066 (5)	−0.0017 (5)
C2	0.0304 (8)	0.0157 (7)	0.0300 (8)	−0.0013 (6)	0.0013 (6)	0.0014 (6)
N6	0.0334 (7)	0.0140 (6)	0.0427 (8)	−0.0005 (5)	0.0080 (6)	−0.0015 (5)
N3	0.0277 (6)	0.0172 (6)	0.0307 (7)	−0.0014 (5)	0.0036 (5)	0.0010 (5)
C4	0.0250 (7)	0.0202 (7)	0.0268 (8)	−0.0008 (5)	0.0001 (6)	0.0017 (6)
S1	0.0273 (2)	0.0258 (2)	0.0404 (3)	0.00045 (14)	0.00645 (16)	0.00140 (15)
C5	0.0268 (7)	0.0155 (7)	0.0292 (8)	0.0015 (5)	0.0020 (6)	0.0003 (5)
C6	0.0281 (7)	0.0151 (6)	0.0293 (8)	0.0011 (5)	0.0024 (6)	0.0004 (5)
O1	0.0321 (6)	0.0147 (5)	0.0395 (6)	−0.0013 (4)	0.0085 (5)	0.0017 (4)
C7	0.0326 (8)	0.0133 (6)	0.0323 (8)	0.0012 (6)	0.0022 (6)	0.0009 (6)
C8	0.0372 (9)	0.0407 (10)	0.0347 (9)	0.0056 (7)	−0.0016 (7)	−0.0074 (7)
C9	0.0293 (8)	0.0177 (7)	0.0357 (8)	0.0006 (6)	0.0024 (6)	0.0042 (6)
C10	0.0386 (10)	0.0376 (10)	0.0398 (10)	−0.0085 (7)	−0.0007 (8)	0.0043 (8)
C11	0.0309 (9)	0.0582 (13)	0.0523 (12)	−0.0121 (8)	−0.0017 (8)	0.0152 (10)
C12	0.0310 (9)	0.0529 (12)	0.0544 (12)	0.0046 (8)	0.0113 (8)	0.0105 (9)
C13	0.0400 (10)	0.0495 (11)	0.0485 (11)	0.0038 (9)	0.0118 (8)	−0.0079 (9)
C14	0.0317 (8)	0.0314 (8)	0.0423 (10)	0.0003 (7)	0.0055 (7)	−0.0062 (7)
N4	0.0328 (7)	0.0215 (7)	0.0404 (8)	0.0024 (5)	0.0059 (6)	0.0005 (6)
C15	0.0254 (7)	0.0206 (7)	0.0310 (8)	−0.0006 (6)	0.0029 (6)	0.0020 (6)
C16	0.0404 (10)	0.0290 (9)	0.0571 (12)	−0.0104 (7)	0.0146 (8)	−0.0067 (8)

### (E)-2-Amino-4-methylsulfanyl-6-oxo-1-(1-phenylethylideneamino)-1,6-dihydropyrimidine-5-carbonitrile (3a) Geometric parameters (Å, º)

**Table:**

N1—C2	1.3705 (19)	C11—C12	1.367 (3)
N1—C6	1.4138 (18)	C12—C13	1.389 (3)
N1—N2	1.4248 (17)	C13—C14	1.382 (3)
N2—C7	1.293 (2)	N4—C15	1.152 (2)
C2—N6	1.326 (2)	N6—H061	0.84 (2)
C2—N3	1.333 (2)	N6—H062	0.84 (2)
N3—C4	1.3343 (19)	C8—H8A	0.9800
C4—C5	1.397 (2)	C8—H8B	0.9800
C4—S1	1.7522 (16)	C8—H8C	0.9800
S1—C16	1.7918 (19)	C10—H10	0.9500
C5—C15	1.424 (2)	C11—H11	0.9500
C5—C6	1.427 (2)	C12—H12	0.9500
C6—O1	1.2253 (18)	C13—H13	0.9500
C7—C9	1.482 (2)	C14—H14	0.9500
C7—C8	1.492 (2)	C16—H16A	0.9800
C9—C10	1.386 (2)	C16—H16B	0.9800
C9—C14	1.401 (2)	C16—H16C	0.9800
C10—C11	1.395 (3)		
			
C2—N1—C6	123.03 (13)	C13—C14—C9	119.84 (17)
C2—N1—N2	117.02 (12)	N4—C15—C5	178.96 (16)
C6—N1—N2	118.81 (11)	C2—N6—H061	120.0 (16)
C7—N2—N1	114.40 (13)	C2—N6—H062	119.2 (17)
N6—C2—N3	120.02 (14)	H061—N6—H062	121 (2)
N6—C2—N1	117.16 (14)	C7—C8—H8A	109.5
N3—C2—N1	122.79 (13)	C7—C8—H8B	109.5
C2—N3—C4	116.77 (13)	H8A—C8—H8B	109.5
N3—C4—C5	124.02 (14)	C7—C8—H8C	109.5
N3—C4—S1	119.47 (11)	H8A—C8—H8C	109.5
C5—C4—S1	116.49 (11)	H8B—C8—H8C	109.5
C4—S1—C16	101.93 (8)	C9—C10—H10	119.7
C4—C5—C15	122.01 (14)	C11—C10—H10	119.7
C4—C5—C6	120.40 (13)	C12—C11—H11	119.8
C15—C5—C6	117.57 (13)	C10—C11—H11	119.8
O1—C6—N1	120.40 (13)	C11—C12—H12	120.3
O1—C6—C5	127.05 (13)	C13—C12—H12	120.3
N1—C6—C5	112.55 (12)	C14—C13—H13	119.5
N2—C7—C9	115.63 (14)	C12—C13—H13	119.5
N2—C7—C8	125.04 (15)	C13—C14—H14	120.1
C9—C7—C8	119.32 (15)	C9—C14—H14	120.1
C10—C9—C14	118.69 (16)	S1—C16—H16A	109.5
C10—C9—C7	120.25 (16)	S1—C16—H16B	109.5
C14—C9—C7	121.04 (15)	H16A—C16—H16B	109.5
C9—C10—C11	120.68 (19)	S1—C16—H16C	109.5
C12—C11—C10	120.41 (18)	H16A—C16—H16C	109.5
C11—C12—C13	119.33 (18)	H16B—C16—H16C	109.5
C14—C13—C12	121.04 (19)		
			
C2—N1—N2—C7	118.94 (15)	N2—N1—C6—C5	−173.87 (13)
C6—N1—N2—C7	−72.88 (18)	C4—C5—C6—O1	−179.25 (16)
C6—N1—C2—N6	−173.38 (14)	C15—C5—C6—O1	2.5 (3)
N2—N1—C2—N6	−5.7 (2)	C4—C5—C6—N1	1.4 (2)
C6—N1—C2—N3	8.5 (2)	C15—C5—C6—N1	−176.89 (14)
N2—N1—C2—N3	176.15 (14)	N1—N2—C7—C9	176.69 (12)
N6—C2—N3—C4	177.46 (15)	N1—N2—C7—C8	−2.8 (2)
N1—C2—N3—C4	−4.5 (2)	N2—C7—C9—C10	−166.23 (15)
C2—N3—C4—C5	−0.7 (2)	C8—C7—C9—C10	13.2 (2)
C2—N3—C4—S1	−179.30 (11)	N2—C7—C9—C14	12.1 (2)
N3—C4—S1—C16	−7.90 (16)	C8—C7—C9—C14	−168.47 (16)
C5—C4—S1—C16	173.40 (13)	C14—C9—C10—C11	−0.5 (3)
N3—C4—C5—C15	−179.67 (15)	C7—C9—C10—C11	177.85 (16)
S1—C4—C5—C15	−1.0 (2)	C9—C10—C11—C12	−0.4 (3)
N3—C4—C5—C6	2.1 (3)	C10—C11—C12—C13	1.0 (3)
S1—C4—C5—C6	−179.25 (12)	C11—C12—C13—C14	−0.7 (3)
C2—N1—C6—O1	174.16 (15)	C12—C13—C14—C9	−0.1 (3)
N2—N1—C6—O1	6.7 (2)	C10—C9—C14—C13	0.7 (3)
C2—N1—C6—C5	−6.5 (2)	C7—C9—C14—C13	−177.59 (16)

### (E)-2-Amino-4-methylsulfanyl-6-oxo-1-(1-phenylethylideneamino)-1,6-dihydropyrimidine-5-carbonitrile (3a) Hydrogen-bond geometry (Å, º)

**Table:**

*D*—H···*A*	*D*—H	H···*A*	*D*···*A*	*D*—H···*A*
N6—H061···O1^i^	0.84 (2)	2.24 (3)	2.9899 (17)	149 (2)
N6—H062···N4^i^	0.84 (2)	2.33 (2)	3.054 (2)	144 (2)
C8—H8*A*···O1^ii^	0.98	2.52	3.279 (2)	134
N6—H062···N2	0.84 (2)	2.25 (2)	2.6273 (19)	108 (2)

Symmetry codes: (i) −*x*+1, *y*+1/2, −*z*+1/2; (ii) *x*, −*y*+1/2, *z*−1/2.

### (E)-2-Amino-4-methylsulfanyl-6-oxo-1-[1-(pyridin-2-yl)ethylideneamino]-1,6-dihydropyrimidine-5-carbonitrile (3b) Crystal data

**Table:**

C_13_H_12_N_6_OS	*F*(000) = 624
*M**_r_* = 300.35	*D*_x_ = 1.460 Mg m^−^^3^
Monoclinic, *P*2_1_/*n*	Cu *K*α radiation, λ = 1.54184 Å
*a* = 13.4774 (5) Å	Cell parameters from 27720 reflections
*b* = 7.6797 (3) Å	θ = 3.9–77.3°
*c* = 14.2755 (6) Å	µ = 2.19 mm^−^^1^
β = 112.401 (5)°	*T* = 100 K
*V* = 1366.04 (10) Å^3^	Plate, orange
*Z* = 4	0.12 × 0.08 × 0.02 mm

### (E)-2-Amino-4-methylsulfanyl-6-oxo-1-[1-(pyridin-2-yl)ethylideneamino]-1,6-dihydropyrimidine-5-carbonitrile (3b) Data collection

**Table:**

Rigaku XtaLAB Synergy, Single source at home/near, HyPix diffractometer	2905 independent reflections
Radiation source: micro-focus sealed X-ray tube	2813 reflections with *I* > 2σ(*I*)
Detector resolution: 10.0000 pixels mm^-1^	*R*_int_ = 0.042
ω scans	θ_max_ = 77.6°, θ_min_ = 3.8°
Absorption correction: multi-scan (CrysAlisPro; Rigaku OD, 2021)	*h* = −17→17
*T*_min_ = 0.782, *T*_max_ = 1.000	*k* = −9→9
54131 measured reflections	*l* = −18→18

### (E)-2-Amino-4-methylsulfanyl-6-oxo-1-[1-(pyridin-2-yl)ethylideneamino]-1,6-dihydropyrimidine-5-carbonitrile (3b) Refinement

**Table:**

Refinement on *F*^2^	Primary atom site location: dual
Least-squares matrix: full	Hydrogen site location: mixed
*R*[*F*^2^ > 2σ(*F*^2^)] = 0.036	H atoms treated by a mixture of independent and constrained refinement
*wR*(*F*^2^) = 0.097	*w* = 1/[σ^2^(*F*_o_^2^) + (0.0483*P*)^2^ + 0.8192*P*] where *P* = (*F*_o_^2^ + 2*F*_c_^2^)/3
*S* = 1.07	(Δ/σ)_max_ = 0.001
2905 reflections	Δρ_max_ = 0.33 e Å^−^^3^
200 parameters	Δρ_min_ = −0.39 e Å^−^^3^
0 restraints	

### (E)-2-Amino-4-methylsulfanyl-6-oxo-1-[1-(pyridin-2-yl)ethylideneamino]-1,6-dihydropyrimidine-5-carbonitrile (3b) Special details

**Table:**

Geometry. All esds (except the esd in the dihedral angle between two l.s. planes) are estimated using the full covariance matrix. The cell esds are taken into account individually in the estimation of esds in distances, angles and torsion angles; correlations between esds in cell parameters are only used when they are defined by crystal symmetry. An approximate (isotropic) treatment of cell esds is used for estimating esds involving l.s. planes. Least-squares planes (x,y,z in crystal coordinates) and deviations from them (* indicates atom used to define plane) - 6.6755 (0.0064) x + 6.5784 (0.0022) y + 0.7860 (0.0078) z = 2.0405 (0.0036) * 0.0570 (0.0009) N1 * -0.0128 (0.0009) C2 * -0.0287 (0.0009) N3 * 0.0218 (0.0009) C4 * 0.0224 (0.0009) C5 * -0.0598 (0.0009) C6 -0.2034 (0.0018) O1 -0.1436 (0.0019) N2 -0.0415 (0.0019) N6 0.0815 (0.0018) S1 -0.1786 (0.0031) C15 Rms deviation of fitted atoms = 0.0383 6.7262 (0.0067) x + 6.5062 (0.0024) y - 0.3110 (0.0086) z = 4.9821 (0.0009) Angle to previous plane (with approximate esd) = 63.117 ( 0.033 ) * -0.0062 (0.0009) C9 * -0.0031 (0.0009) N5 * 0.0095 (0.0010) C10 * -0.0064 (0.0011) C11 * -0.0026 (0.0011) C12 * 0.0087 (0.0010) C13 Rms deviation of fitted atoms = 0.0066

### (E)-2-Amino-4-methylsulfanyl-6-oxo-1-[1-(pyridin-2-yl)ethylideneamino]-1,6-dihydropyrimidine-5-carbonitrile (3b) Fractional atomic coordinates and isotropic or equivalent isotropic displacement parameters (Å^2^)

**Table:**

	*x*	*y*	*z*	*U*_iso_*/*U*_eq_	
N1	0.24070 (9)	0.52162 (15)	0.34716 (8)	0.0181 (2)	
N2	0.25298 (10)	0.51485 (15)	0.25281 (9)	0.0200 (2)	
C2	0.32652 (10)	0.58859 (17)	0.42667 (10)	0.0180 (3)	
N6	0.40369 (9)	0.66515 (16)	0.40482 (9)	0.0210 (2)	
H061	0.4563 (16)	0.711 (3)	0.4535 (15)	0.034 (5)*	
H062	0.3927 (14)	0.680 (2)	0.3424 (15)	0.026 (4)*	
N3	0.33350 (9)	0.58187 (16)	0.52197 (8)	0.0202 (2)	
C4	0.25208 (11)	0.50497 (18)	0.53832 (10)	0.0211 (3)	
S1	0.25540 (3)	0.50264 (6)	0.66187 (3)	0.03285 (13)	
C5	0.16475 (10)	0.42545 (18)	0.46300 (10)	0.0202 (3)	
C6	0.15960 (10)	0.41990 (17)	0.36111 (10)	0.0186 (3)	
O1	0.09466 (7)	0.34075 (13)	0.28923 (7)	0.0216 (2)	
C7	0.17715 (10)	0.58645 (17)	0.17779 (10)	0.0182 (3)	
C8	0.08130 (10)	0.68105 (18)	0.17933 (10)	0.0201 (3)	
H8A	0.016282	0.615319	0.140383	0.030*	
H8B	0.077686	0.796660	0.149146	0.030*	
H8C	0.086858	0.693400	0.249489	0.030*	
C9	0.19145 (10)	0.57067 (17)	0.07948 (10)	0.0189 (3)	
N5	0.11472 (9)	0.64664 (16)	−0.00075 (8)	0.0220 (3)	
C10	0.12509 (11)	0.6336 (2)	−0.09027 (11)	0.0259 (3)	
H10	0.072632	0.688318	−0.147422	0.031*	
C11	0.20768 (12)	0.5451 (2)	−0.10409 (11)	0.0279 (3)	
H11	0.210358	0.537126	−0.169500	0.034*	
C12	0.28675 (12)	0.4679 (2)	−0.02112 (12)	0.0262 (3)	
H12	0.344761	0.406811	−0.028270	0.031*	
C13	0.27859 (11)	0.48252 (18)	0.07219 (11)	0.0227 (3)	
H13	0.331879	0.432958	0.130782	0.027*	
C14	0.08272 (11)	0.33814 (19)	0.48529 (10)	0.0227 (3)	
N4	0.01756 (10)	0.26795 (19)	0.50465 (10)	0.0308 (3)	
C15	0.38704 (14)	0.5880 (3)	0.73425 (12)	0.0390 (4)	
H15A	0.393874	0.704706	0.709543	0.058*	
H15B	0.441562	0.511218	0.726668	0.058*	
H15C	0.397265	0.594636	0.805906	0.058*	

### (E)-2-Amino-4-methylsulfanyl-6-oxo-1-[1-(pyridin-2-yl)ethylideneamino]-1,6-dihydropyrimidine-5-carbonitrile (3b) Atomic displacement parameters (Å^2^)

**Table:**

	*U*^11^	*U*^22^	*U*^33^	*U*^12^	*U*^13^	*U*^23^
N1	0.0166 (5)	0.0247 (6)	0.0125 (5)	0.0003 (4)	0.0049 (4)	0.0006 (4)
N2	0.0198 (5)	0.0264 (6)	0.0136 (5)	0.0002 (4)	0.0064 (4)	−0.0001 (4)
C2	0.0157 (6)	0.0207 (6)	0.0162 (6)	0.0028 (5)	0.0045 (5)	0.0002 (5)
N6	0.0180 (5)	0.0300 (6)	0.0141 (5)	−0.0032 (5)	0.0051 (4)	−0.0004 (5)
N3	0.0187 (5)	0.0270 (6)	0.0146 (5)	−0.0012 (4)	0.0059 (4)	0.0002 (4)
C4	0.0206 (6)	0.0266 (7)	0.0161 (6)	−0.0004 (5)	0.0071 (5)	0.0005 (5)
S1	0.0295 (2)	0.0535 (3)	0.0176 (2)	−0.01433 (17)	0.01126 (16)	−0.00596 (15)
C5	0.0181 (6)	0.0250 (7)	0.0177 (6)	−0.0004 (5)	0.0068 (5)	0.0009 (5)
C6	0.0153 (6)	0.0214 (6)	0.0172 (6)	0.0028 (5)	0.0040 (5)	0.0017 (5)
O1	0.0189 (4)	0.0266 (5)	0.0164 (4)	−0.0019 (4)	0.0034 (4)	−0.0010 (4)
C7	0.0172 (6)	0.0201 (6)	0.0162 (6)	−0.0032 (5)	0.0050 (5)	−0.0006 (5)
C8	0.0185 (6)	0.0232 (6)	0.0173 (6)	0.0008 (5)	0.0053 (5)	0.0010 (5)
C9	0.0183 (6)	0.0204 (6)	0.0171 (6)	−0.0035 (5)	0.0059 (5)	−0.0020 (5)
N5	0.0177 (5)	0.0300 (6)	0.0164 (5)	−0.0025 (5)	0.0042 (4)	0.0001 (4)
C10	0.0208 (6)	0.0387 (8)	0.0156 (6)	−0.0042 (6)	0.0041 (5)	0.0003 (6)
C11	0.0279 (7)	0.0399 (8)	0.0175 (6)	−0.0050 (6)	0.0102 (6)	−0.0047 (6)
C12	0.0254 (7)	0.0300 (7)	0.0262 (7)	−0.0011 (6)	0.0133 (6)	−0.0040 (6)
C13	0.0223 (7)	0.0239 (7)	0.0215 (7)	0.0002 (5)	0.0077 (5)	0.0012 (5)
C14	0.0208 (6)	0.0270 (7)	0.0191 (6)	−0.0003 (5)	0.0061 (5)	0.0000 (5)
N4	0.0256 (6)	0.0353 (7)	0.0330 (7)	−0.0034 (5)	0.0128 (5)	0.0028 (6)
C15	0.0344 (8)	0.0639 (12)	0.0189 (7)	−0.0177 (8)	0.0104 (6)	−0.0091 (7)

### (E)-2-Amino-4-methylsulfanyl-6-oxo-1-[1-(pyridin-2-yl)ethylideneamino]-1,6-dihydropyrimidine-5-carbonitrile (3b) Geometric parameters (Å, º)

**Table:**

N1—C2	1.3747 (17)	N5—C10	1.3412 (18)
N1—C6	1.4179 (17)	C10—C11	1.381 (2)
N1—N2	1.4191 (16)	C11—C12	1.389 (2)
N2—C7	1.2883 (18)	C12—C13	1.382 (2)
C2—N3	1.3286 (17)	C14—N4	1.1503 (19)
C2—N6	1.3312 (17)	N6—H061	0.86 (2)
N3—C4	1.3425 (17)	N6—H062	0.853 (19)
C4—C5	1.3963 (19)	C8—H8A	0.9800
C4—S1	1.7473 (14)	C8—H8B	0.9800
S1—C15	1.8023 (16)	C8—H8C	0.9800
C5—C14	1.4287 (18)	C10—H10	0.9500
C5—C6	1.4303 (18)	C11—H11	0.9500
C6—O1	1.2263 (16)	C12—H12	0.9500
C7—C8	1.4896 (18)	C13—H13	0.9500
C7—C9	1.4925 (17)	C15—H15A	0.9800
C9—N5	1.3486 (17)	C15—H15B	0.9800
C9—C13	1.3934 (19)	C15—H15C	0.9800
			
C2—N1—C6	122.76 (11)	C13—C12—C11	118.05 (14)
C2—N1—N2	115.57 (10)	C12—C13—C9	119.36 (13)
C6—N1—N2	119.21 (10)	N4—C14—C5	179.05 (15)
C7—N2—N1	115.53 (11)	C2—N6—H061	118.1 (13)
N3—C2—N6	120.03 (12)	C2—N6—H062	117.7 (12)
N3—C2—N1	122.68 (12)	H061—N6—H062	123.4 (18)
N6—C2—N1	117.28 (12)	C7—C8—H8A	109.5
C2—N3—C4	116.84 (11)	C7—C8—H8B	109.5
N3—C4—C5	124.29 (12)	H8A—C8—H8B	109.5
N3—C4—S1	118.15 (10)	C7—C8—H8C	109.5
C5—C4—S1	117.56 (10)	H8A—C8—H8C	109.5
C4—S1—C15	102.47 (7)	H8B—C8—H8C	109.5
C4—C5—C14	122.04 (12)	N5—C10—H10	118.1
C4—C5—C6	119.75 (12)	C11—C10—H10	118.1
C14—C5—C6	118.08 (12)	C10—C11—H11	120.4
O1—C6—N1	119.88 (12)	C12—C11—H11	120.4
O1—C6—C5	127.47 (12)	C13—C12—H12	121.0
N1—C6—C5	112.65 (11)	C11—C12—H12	121.0
N2—C7—C8	127.85 (12)	C12—C13—H13	120.3
N2—C7—C9	113.66 (12)	C9—C13—H13	120.3
C8—C7—C9	118.49 (11)	S1—C15—H15A	109.5
N5—C9—C13	122.82 (12)	S1—C15—H15B	109.5
N5—C9—C7	115.56 (12)	H15A—C15—H15B	109.5
C13—C9—C7	121.62 (12)	S1—C15—H15C	109.5
C10—N5—C9	116.89 (12)	H15A—C15—H15C	109.5
N5—C10—C11	123.73 (13)	H15B—C15—H15C	109.5
C10—C11—C12	119.12 (13)		
			
C2—N1—N2—C7	−127.19 (13)	N2—N1—C6—C5	173.52 (11)
C6—N1—N2—C7	70.10 (15)	C4—C5—C6—O1	172.14 (13)
C6—N1—C2—N3	−8.6 (2)	C14—C5—C6—O1	−3.8 (2)
N2—N1—C2—N3	−170.61 (12)	C4—C5—C6—N1	−8.43 (18)
C6—N1—C2—N6	172.59 (12)	C14—C5—C6—N1	175.67 (12)
N2—N1—C2—N6	10.56 (17)	N1—N2—C7—C8	2.8 (2)
N6—C2—N3—C4	179.06 (12)	N1—N2—C7—C9	−177.94 (10)
N1—C2—N3—C4	0.26 (19)	N2—C7—C9—N5	−178.88 (12)
C2—N3—C4—C5	3.3 (2)	C8—C7—C9—N5	0.50 (17)
C2—N3—C4—S1	−177.08 (10)	N2—C7—C9—C13	1.27 (18)
N3—C4—S1—C15	−7.55 (14)	C8—C7—C9—C13	−179.35 (12)
C5—C4—S1—C15	172.11 (12)	C13—C9—N5—C10	0.3 (2)
N3—C4—C5—C14	176.96 (13)	C7—C9—N5—C10	−179.57 (12)
S1—C4—C5—C14	−2.67 (19)	C9—N5—C10—C11	1.3 (2)
N3—C4—C5—C6	1.2 (2)	N5—C10—C11—C12	−1.6 (2)
S1—C4—C5—C6	−178.41 (10)	C10—C11—C12—C13	0.4 (2)
C2—N1—C6—O1	−168.41 (12)	C11—C12—C13—C9	1.0 (2)
N2—N1—C6—O1	−7.00 (18)	N5—C9—C13—C12	−1.4 (2)
C2—N1—C6—C5	12.11 (17)	C7—C9—C13—C12	178.41 (12)

### (E)-2-Amino-4-methylsulfanyl-6-oxo-1-[1-(pyridin-2-yl)ethylideneamino]-1,6-dihydropyrimidine-5-carbonitrile (3b) Hydrogen-bond geometry (Å, º)

**Table:**

*D*—H···*A*	*D*—H	H···*A*	*D*···*A*	*D*—H···*A*
N6—H061···N5^i^	0.86 (2)	2.26 (2)	3.0122 (17)	146.2 (17)
N6—H062···O1^ii^	0.853 (19)	2.307 (19)	3.0886 (15)	152.4 (17)
C10—H10···O1^iii^	0.95	2.40	3.2351 (17)	147
N6—H062···N2	0.853 (19)	2.228 (18)	2.6091 (17)	107.1 (14)

Symmetry codes: (i) *x*+1/2, −*y*+3/2, *z*+1/2; (ii) −*x*+1/2, *y*+1/2, −*z*+1/2; (iii) −*x*, −*y*+1, −*z*.
